# Impact of Procedure Volumes and Focused Practice on Short-Term Outcomes of Elective and Urgent Colon Cancer Resection in Italy

**DOI:** 10.1371/journal.pone.0064245

**Published:** 2013-05-16

**Authors:** Jacopo Lenzi, Raffaele Lombardi, Davide Gori, Nicola Zanini, Dario Tedesco, Michele Masetti, Elio Jovine, Maria Pia Fantini

**Affiliations:** 1 Department of Biomedical and Neuromotor Sciences, Alma Mater Studiorum - University of Bologna, Bologna, Italy; 2 General Surgery Unit, Department of Surgery, Maggiore Hospital, Bologna, Italy; University of Bari & Consorzio Mario Negri Sud, Italy

## Abstract

**Background:**

The relationship between hospital volumes and short-term patients’ outcomes of colon cancer (CC) surgery is not well established in the literature. Moreover, evidence about short-term outcomes of urgent compared with elective CC procedures is scanty. The aims of this study are 1) to determine whether caseloads and other hospital characteristics are associated with short-term outcomes of CC surgery; 2) to compare the outcomes of urgent and elective CC surgery.

**Methods:**

A total of 14,200 patients undergoing CC surgery between 2005 and 2010 in the General Surgery Units (GSUs) of the hospitals of Emilia-Romagna region, Northern Italy, were identified from the hospital discharge records database. The outcomes of interest were 30-day in-hospital mortality, re-intervention and 30-day re-admission. Using multilevel analysis, we analyzed the relationship of GSU volumes and focused practice, defined as the percentage of CC operations over total operations, with the three outcomes.

**Results:**

High procedure volumes were associated with a lower risk of 30-day in-hospital mortality, after adjusting for patients’ characteristics [aOR (95% CI) = 0.51 (0.33–0.81)]. Stratified analyses for elective and urgent surgery showed that high volumes were associated with a lower 30-day mortality for elective patients [aOR (95% CI) = 0.35 (0.17–0.71)], but not for urgent patients [aOR (95% CI) = 0.72 (0.42–1.24)]. Focused practice was an independent predictor of re-intervention [aOR (95% CI) = 0.67 (0.47–0.97)] and re-admission [aRR (95% CI) = 0.88 (0.78–0.98)].

**Conclusions:**

The present study adds evidence in support of the notion that patients with CC undergoing surgery at high-volume and focused surgical units experience better short-term outcomes.

## Introduction

In Western countries, colorectal cancer is the third most commonly diagnosed cancer in males and the second in females. About two-thirds of colorectal cancers occur in the colon [1] and early diagnosis and radical resection may represent the only chance of cure for patients [2]. This has led many Western countries, including Italy, to introduce colon cancer (CC) screening programs. In 2005 Emilia-Romagna region, in Northern Italy, launched a screening program for early detection of colorectal cancer targeted to people aged 50–74 years, with a compliance of 46.7% in 2007 that increased to 53.7% in 2008 [3].

Despite substantial advances in surgical techniques and peri-operative care during the last decades, morbidity and mortality after surgery remain considerable, ranging from 18% to 35% and 1% to 11%, respectively [4]–[8]. However, it is well known that the risk of adverse events after colorectal surgery depends on patient-, disease-, and treatment-related characteristics, some of which are modifiable [9], [10]. Moreover, identification of outcome predictors liable to preventive measures is crucial for improving surgical care quality.

Since late 1970s, several authors analyzed the relationship between hospital volume and short- and long-term outcomes, and found a positive correlation for complex surgical procedures [11]–[17]. A recent Cochrane review and meta-analysis based on studies carried out in USA, UK and Northern Europe showed that higher surgeon volumes were associated with better outcomes of CC surgery, while hospital volumes were unrelated with these outcomes [18].

To our knowledge, no study has investigated the relationship between caseloads and outcomes of CC surgery in Italy, where colorectal surgery is performed in General Surgery Units (GSUs). Moreover, little is known about the outcomes of CC surgery in elective and urgent patients. In a recent study carried out in Denmark, the authors found a significant variation in mortality between low- and high-volume hospitals for urgent surgery, but not for elective surgery [19].

The aims of the present study are: 1) to determine whether caseloads and other hospital characteristics are associated with short-term outcomes of CC surgery; 2) to compare the outcomes of urgent and elective CC surgery.

## Materials and Methods

### Ethics Statement

The study was carried out in conformity with the regulations on data management of the Regional Health Authority of Emilia-Romagna, and with the Italian law on privacy (Art. 20–21, DL 196/2003) (http://www.garanteprivacy.it/web/guest/home/docweb/-/docweb-display/docweb/1115480, published in the Official Journal no. 190 of August 14, 2004) which explicitly exempts the need of ethical approval for anonymous data (Preamble #8).

Data were anonymized prior to the analysis at the regional statistical office, where each patient was assigned a unique identifier. This identifier does not allow to trace the patient’s identity and other sensitive data. As anonymized administrative data are used routinely for health-care management no specific written informed consent was needed to use patient information.

The data set will be made freely available upon request.

### Population and Data

Data were extracted from the Hospital Discharge Records (HDRs) database, that includes all discharges from the 86 GSUs of the 66 hospitals in Emilia-Romagna region (4.4 million inhabitants, 42% aged >50 years) [20]. GSUs provide, in addition to gastrointestinal surgery, abdominal, thyroid and breast surgery. Large hospitals may have more than one GSU.

For each GSU, volume was defined as the mean annual number of CC procedures carried out over 6 years, and focused practice as the percentage of CC operations over total operations. A tertile split was used to classify GSUs into three volume categories: low-volume (<40 CC cases/year), intermediate-volume (40–64 CC cases/year), or high-volume (≥65 CC cases/year). A median split was used to classify GSUs as non-focused (<5% CC cases over total operations) or focused (≥5% CC cases).

Hospitals were categorized as private or public and teaching or non-teaching. Public hospitals are owned by the regional government, while private hospitals are privately owned. In the presence of an agreement with the Regional Health Authority, private hospitals supply services for the regional health care system and receive public funding. Teaching hospitals are public hospitals affiliated with a medical school.

ICD-9-CM codes were used to identify patients with a primary diagnosis of carcinoma *in situ* or malignant neoplasm of the colon (codes 230.3 and 153.x, respectively) and an operation in the digestive system (codes 42–54) as the primary procedure. This methodology decreases the risk of excluding from the analyses patients undergoing multi-visceral resections for locally advanced colonic tumors. 14,809 HDRs were extracted for the period 1/1/2005–12/31/2010. 609 transfers from other hospitals were excluded.

Independent variables used for case mix-adjusted analyses were: age, sex, length of stay of the index admission, comorbidities, presence/absence of metastases, type of resection and type of admission (urgent/elective). Comorbidity was assessed using secondary diagnoses at the index admission and in the two previous years. Tumor spread was determined using diagnostic codes that signaled the involvement of other organs (197.x and 198.89). In the absence of these codes, it was assumed that no metastasis was present. Interventions were categorized as partial colectomies (code 45.7) or total colectomies (code 45.8). The remaining interventions were classified as “other”.

### Outcome Measures

The outcomes considered were: 30-day mortality (death within 30 days of surgery related to the index or any subsequent hospitalization), 30-day re-admission (admission occurring for any reason within 30 days of index discharge) and re-intervention in the index hospitalization, identified by means of a specific algorithm that combines surgical procedures and complications occurring in the days after CC surgery (Text S1).

### Statistical Analysis

Student’s *t*-test, *χ^2^* test and Fisher’s exact test were used, where appropriate, to analyze the relationship between patient characteristics and each of the three outcomes. We also analyzed the relationship between GSU volume and focused practice using Spearman’s *rho*.

In order to allow for the hierarchical structure of the data, in which patients are clustered into GSUs and GSUs into hospitals, we analyzed the relationship of GSU and hospital characteristics with outcomes using multilevel logistic regression analyses. For each outcome, the multilevel analysis was carried out in two steps. In the first step, a three-level model (M1) was built including patient characteristics significantly (*p*<0.05) associated with the outcome and random intercepts for GSUs and hospitals. In the second step, significant GSU and hospital characteristics were added to the model (M2) to determine the variability in outcome associated with these variables after controlling for patient case mix. In this model, we also tested the presence of interactions between GSU and hospital characteristics and the admission status (elective/urgent).

We present the associations of GSUs and hospital characteristics with outcomes deriving from the model M2 in terms of odds ratios (ORs) or risk ratios (RRs) with 95% confidence intervals (95% CIs) [21]. We also provide GSU- and hospital-level variance of the model M2, and how much of this variability is attributable to GSU and hospital characteristics. This last measure is calculated as the proportional change in variance between M1 and M2.

Statistical analyses were carried out using the procedure xtmelogit of Stata software, version 12 (StataCorp. 2011. *Stata Statistical Software:* Release 12. College Station, TX: StataCorp LP).

## Results

### Baseline Characteristics and Population Case-mix

The study cohort consisted of 14,200 patients: 7,722 men (54.4%), with a mean age of 70 years. A total of 10,831 patients underwent elective operation (76.27%) and 3,369 patients urgent operation (23.73%) (Table 1).

**Table 1:** Patient characteristics.

Study sample	*n* = 14,200
**Age in years, mean [SD]**	70.16 [11.34]
**Gender (%)**	
Male	7,722 (54.38)
Female	6,478 (45.62)
**Length of stay in days, median [IQR]**	11 [7]
**Comorbidities (%)**	
No	6,328 (44.56)
Yes	7,872 (55.44)
**Specific comorbidities (%)**	
Diabetes	1,370 (9.65)
Disorders of lipoid metabolism	295 (2.08)
Hematologic diseases	2,484 (17.49)
Hypertensive disease	1,845 (12.99)
Old acute myocardial infarction	312 (2.20)
Other forms of ischemic heart disease	707 (4.98)
Heart failure	289 (2.04)
Ill-defined descriptions and complications of heart disease	84 (0.59)
Rheumatic heart disease	129 (0.91)
Cardiomyopathies	88 (0.62)
Acute endocarditis and myocarditis	7 (0.05)
Other cardiac diseases	211 (1.49)
Conduction disorders and cardiac dysrhythmias	613 (4.32)
Cerebrovascular diseases	593 (4.18)
Vascular diseases	403 (2.84)
Chronic obstructive pulmonary disease (COPD)	1,149 (8.09)
Chronic nephropathies	296 (2.08)
Chronic diseases (liver, pancreas and intestine)	360 (2.54)
History of tumors	6,126 (43.14)
Tumors other than colorectal cancer at theindex admission	113 (0.80)
**Presence of metastases (%)**	
No	11,899 (83.80)
Yes	2,301 (16.20)
**Type of procedure (%)** a	
Partial colectomy	13,424 (94.59)
Total colectomy	268 (1.89)
Other	499 (3.52)
**Admission status (%)**	
Elective	10,831 (76.27)
Urgent	3,369 (23.73)

a Patients with unknown type of procedure were not included (n = 9).

SD, standard deviation; IQR, interquartile range.

Of the 66 hospitals included in the present analyses, 4 were teaching and 62 non-teaching hospitals (93.9% of hospitals), 23 were private and 43 public (65.2% of hospitals). Twelve hospitals had more than one GSU. Of these, eight hospitals had two GSUs, and four hospitals had more than two GSUs.

Of the nine high-volume GSUs, six operated in non-teaching public hospitals and three in teaching hospitals; private hospitals had only low-volume GSUs. Of the twenty-two focused GSUs, more than half (twelve) operated in non-teaching public hospitals (Table 2).

**Table 2:** GSU and hospital characteristics.

GSU volume	Non-teaching public hospitals (*n* = 39)		Teaching public hospitals (*n* = 4)		Private hospitals (*n* = 23)		
	Non-focused GSUs (%)	Focused GSUs^a^ (%)	Non-focused GSUs (%)	Focused GSUs^a^ (%)	Non-focused GSUs (%)	Focused GSUs^a^ (%)	
Low-volume (<40)	26 (74.3)	2 (16.6)	5 (71.4)	3 (37.5)	22 (100.0)	2 (100.0)	
Intermediate-volume (40–64)	8 (22.9)	5 (41.7)	0 (0.0)	4 (50.0)	0 (0.0)	0 (0.0)	
High-volume (≥65)	1 (2.9)	5 (41.7)	2 (28.6)	1 (12.5)	0 (0.0)	0 (0.0)	
Total	35 (100.0)	12 (100.0)	7 (100.0)	8 (100.0)	22 (100.0)	2 (100.0)	

a Defined as GSUs with over 5% CC cases over total operations.

GSU, General surgery unit.

GSU volume and focused practice were moderately correlated (Spearman’s *rho* = 0.49, *p*<0.001), suggesting that the two variables are not interchangeable.

### Outcomes

The prevalence of 30-day in-hospital mortality, 30- day re-admission, and re-intervention was 1.9% (range, 0.0%–16.7%), 28.1% (range, 0.0%–60.0%), and 3.3% (range, 0.0%–14.3%), respectively.

### Crude Associations of Patient Characteristics with Outcomes

Crude associations of patient characteristics with outcomes are shown in Table 3. 30-day mortality was significantly higher among patients with at least one comorbidity [2.5% *vs.* 1.2%, *p*<0.001; OR (95% CI) = 2.05 (1.57–2.67)], and among those who underwent urgent procedures [5.2% *vs.* 0.9%, *p*<0.001; OR (95% CI) = 5.84 (4.56–7.50)]. The same associations were found for re-interventions; re-admission was more likely among younger patients and among those undergoing urgent surgery.

**Table 3:** Crude relationships of patient characteristics with outcomes.

Characteristics	30-day mortality			Re-intervention			30-day re-admission		
	No(*n* = 13,926)	Yes(*n* = 274)	*p*-value	No(*n* = 13,732)	Yes(*n* = 468)	*p*-value	No(*n* = 10,209)	Yes(*n* = 3,991)	*p*-value
**Age in years, mean [SD]**	69.93 [11.27]	81.72 [8.55]	<0.001	70.10 [11.29]	71.80 [12.51]	0.001	71.23 [11.05]	67.42 [11.60]	<0.001
**Gender (%)**			0.540			0.540			0.021
Male	7,578 (54.42)	144 (52.55)		7,461 (54.33)	261 (55.77)		5,490 (53.78)	2,232 (55.93)	
Female	6,348 (45.58)	130 (47.45)		6,271 (45.67)	207 (44.23)		4,719 (46.22)	1,759 (44.07)	
**Comorbidities (%)**			<0.001			0.016			0.102
No	6,250 (44.88)	78 (28.47)		6,145 (44.75)	183 (39.10)		4,506 (44.14)	1,822 (45.65)	
Yes	7,676 (55.12)	196 (71.53)		7,587 (55.25)	285 (60.90)		5,703 (55.86)	2,169 (54.35)	
**Specific comorbidities (%)**									
Diabetes	1,343 (9.64)	27 (9.85)	0.907	1,320 (9.61)	50 (10.68)	0.440	1,024 (10.03)	346 (8.67)	0.014
Disorders of lipoid metabolism	285 (2.05)	10 (3.65)	0.065	289 (2.10)	6 (1.28)	0.220	209 (2.05)	86 (2.15)	0.686
Hematologic diseases	2,411 (17.31)	73 (26.64)	<0.001	2,343 (17.06)	141 (30.13)	<0.001	1,794 (17.57)	690 (17.29)	0.689
Hypertensive disease	1,782 (12.80)	63 (22.99)	<0.001	1,783 (12.98)	62 (13.25)	0.868	1,376 (13.48)	469 (11.75)	0.006
Old acute myocardial infarction	304 (2.18)	8 (2.92)	0.410	306 (2.23)	6 (1.28)	0.170	244 (2.39)	68 (1.70)	0.012
Other forms of ischemic heartdisease	672 (4.83)	35 (12.77)	<0.001	682 (4.97)	25 (5.34)	0.714	533 (5.22)	174 (4.36)	0.034
Heart failure	267 (1.92)	22 (8.03)	<0.001	280 (2.04)	9 (1.92)	0.861	228 (2.23)	61 (1.53)	0.007
Ill-defined descriptionsand complications ofheart disease	78 (0.56)	6 (2.19)	0.006	82 (0.60)	2 (0.43)	1.000	66 (0.65)	18 (0.45)	0.172
Rheumatic heart disease	123 (0.88)	6 (2.19)	0.039	123 (0.90)	6 (1.28)	0.323	99 (0.97)	30 (0.75)	0.218
Cardiomyopathies	82 (0.59)	6 (2.19)	0.007	84 (0.61)	4 (0.85)	0.539	69 (0.68)	19 (0.48)	0.173
Acute endocarditis and myocarditis	7 (0.05)	0 (0.00)	1.000	6 (0.04)	1 (0.21)	0.209	6 (0.06)	1 (0.03)	0.681
Other cardiac diseases	204 (1.46)	7 (2.55)	0.131	195 (1.42)	16 (3.42)	<0.001	165 (1.62)	46 (1.15)	0.040
Conduction disorders and cardiac dysrhythmias	581 (4.17)	32 (11.68)	<0.001	584 (4.25)	29 (6.20)	0.042	454 (4.45)	159 (3.98)	0.222
Cerebrovascular diseases	557 (4.00)	36 (13.14)	<0.001	574 (4.18)	19 (4.06)	0.898	444 (4.35)	149 (3.73)	0.099
Vascular diseases	378 (2.71)	25 (9.12)	<0.001	389 (2.83)	14 (2.99)	0.839	322 (3.15)	81 (2.03)	<0.001
COPD	1,100 (7.90)	49 (17.88)	<0.001	1,107 (8.06)	42 (8.97)	0.476	866 (8.48)	283 (7.09)	0.006
Chronic nephropathies	277 (1.99)	19 (6.93)	<0.001	288 (2.10)	8 (1.71)	0.564	228 (2.23)	68 (1.70)	0.047
Chronic diseases (liver,pancreas and intestine)	348 (2.50)	12 (4.38)	0.050	351 (2.56)	9 (1.92)	0.392	263 (2.58)	97 (2.43)	0.620
History of tumors	5,961 (42.80)	165 (60.22)	<0.001	5,929 (43.18)	197 (42.09)	0.642	4,417 (43.27)	1,709 (42.82)	0.631
Tumors other thancolorectal cancer atthe index admission	110 (0.79)	3 (1.09)	0.482	108 (0.79)	5 (1.07)	0.425	74 (0.72)	39 (0.98)	0.128
**Presence of metastases (%)**			<0.001			0.154			<0.001
No	11,712 (84.10)	187 (68.25)		11,518 (83.88)	381 (81.41)		8,919 (87.36)	2,980 (74.67)	
Yes	2,214 (15.90)	87 (31.75)		2,214 (16.12)	87 (18.59)		1,290 (12.64)	1,011 (25.33)	
**Type of procedure (%)** a			<0.001			0.001			0.001
Partial colectomy	13,196 (94.81)	228 (83.52)		12,993 (94.68)	431 (92.10)		9,693 (94.98)	3,731 (93.60)	
Total colectomy	261 (1.88)	7 (2.56)		262 (1.91)	6 (1.28)		188 (1.84)	80 (2.01)	
Other	461 (3.31)	38 (13.92)		468 (3.41)	31 (6.62)		324 (3.18)	175 (4.39)	
**Admission status (%)**			<0.001			<0.001			<0.001
Elective	10,731 (77.06)	100 (36.50)		10,602 (77.21)	229 (48.93)		8,004 (78.40)	2,827 (70.83)	
Urgent	3,195 (22.94)	174 (63.50)		3,130 (22.79)	239 (51.07)		2,205 (21.60)	1,164 (29.17)	

a Patients with unknown type of procedure were not included (n = 9).

### Adjusted Associations of GSU and Hospital Characteristics with Outcomes

After adjusting for patient characteristics in multilevel logistic regression analysis (models M2), GSU volume predicted only 30-day mortality. Specifically, patients who underwent surgery at high-volume GSUs had a significant reduction in the mortality risk [aOR (95% CI) = 0.51 (0.33–0.81)] compared with patients undergoing surgery at low-volume GSUs. The mortality risk did not differ significantly between patients who underwent surgery at low- and intermediate-volume GSUs [aOR (95% CI) = 0.83 (0.54–1.27)]. Because of the interaction between admission status (elective/urgent) and GSU volume, a stratified analysis by admission status was carried out: high volumes were associated with a lower 30-day mortality for elective patients [aOR (95% CI) = 0.35 (0.17–0.71)], but not for urgent patients [aOR (95% CI) = 0.72 (0.42–1.24)] (Table 4).

**Table 4:** Multilevel logistic regression analysis: adjusted relationships of GSU characteristics with outcomes.

GSU characteristics	30-day mortality (elective pts)			30-day mortality (urgent pts)			Re-intervention			30-day re-admission		
	aOR^a^	*p*-value	95% CI	aOR^a^	*p*-value	95% CI	aOR^b^	*p*-value	95% CI	aRRc,d	*p*-value	95% CI
**GSU volume**												
Low-volume (<40)	1			1								
Intermediate-volume (40–64)	0.566	0.073	(0.304–1.055)	1.025	0.916	(0.645–1.630)	–			–		
High-volume (≥65)	0.352	0.004	(0.174–0.713)	0.723	0.239	(0.421–1.241)	–			–		
**GSU focused practice**												
Non-focused (<5%)							1			1		
Focused (≥5%)	–			–			0.673	0.034	(0.467–0.971)	0.875	0.044	(0.780–0.981)
Pseudo *R^2^* e	0.429			0.367			0.305			0.129		

a Adjusted for significant patient-level covariates, including sex, age, cardiomyopathies, heart failure, COPD, chronic diseases (liver, pancreas, intestine), vascular diseases, cerebrovascular diseases, history of tumors, admission status, type of procedure and presence of metastases.

b Adjusted for significant patient-level covariates, including sex, age, hematologic diseases, other cardiac diseases, old acute myocardial infarction, admission status and type of procedure.

c Adjusted for significant patient-level covariates, including sex, age, length of stay, diabetes, other cardiac diseases, hematologic diseases, other cardiac diseases, vascular diseases, cerebrovascular diseases, history of tumors, admission status and presence of metastases.

d 30-day re-admission was a common outcome and the OR is not a good approximation to the RR, so we estimated the RR of re-admission using Flanders and Rhodes method [21].

e Pseudo *R^2^* indicates how much of the total variation of the phenomenon (patient-, GSU- and hospital-level variance) was explained by the covariates included in the model.

pts, patients; OR, odds ratio; CI, confidence interval; RR, relative risk.

GSU focused practice was an independent predictor of re-intervention and re-admission. In particular, patients who underwent surgery at focused GSUs had a significant reduction in the risk of re-intervention [aOR (95% CI) = 0.67 (0.47–0.97)] and re-admission [aRR (95% CI) = 0.88 (0.78–0.98)] (Table 4). There was no evidence of an interaction between GSU focused practice and the admission status.

Hospital characteristics (teaching/non-teaching, public/private) were unrelated to the three outcomes.

### Variations among GSUs in 30-day Mortality, Re-intervention and 30-day Re-admission

The random part of the models M2 is shown in Table 5. We found significant variations among GSUs in 30-day mortality after elective surgery (GSU- and hospital-level variance = 0.471; *p* = 0.002) and no significant variations in mortality after urgent surgery (GSU- and hospital-level variance = 0.209; *p* = 0.067). We also found significant variations among GSUs in re-intervention and 30-day re-admission.

**Table 5:** Multilevel logistic regression analysis: variations among GSUs in 30-day mortality, re-intervention and 30-day re-admission.

	30-day mortality(elective pts)	30-day mortality(urgent pts)	Re-intervention	30-day re-admission
**GSU- and hospital-level variance** a	0.471	0.209	0.634	0.470
***p*** **-value** b	0.002	0.067	<0.001	<0.001
**PCV (%)** c	42.07	0.00	7.45	6.00

a GSU- and hospital-level variance is a measure of GSU variations in mortality, re-intervention and re-admission. It is calculated as the sum of hospital-level variance and GSU-level variance, both computed using the restricted maximum likelihood method.

b Determined via likelihood-ratio test of variance = 0.

c PCV (proportional change in variance) is calculated as the percentage decrease between the variance of model M1 and the variance of model M2. It measures the percentage variance attributable to GSU characteristics.

More than 40% of the variability in 30-day mortality after elective surgery was attributable to GSU volume, and GSU focused practice accounted for about 7% and 6% of the differences among GSUs in re-intervention and 30-day re-admission, respectively.

## Discussion

Our results indicate that patients undergoing CC surgery at higher volumes GSUs had a decreased risk of post-operative mortality. This adds to the growing body of evidence (including the recent Cochrane review and meta-analysis [18]) showing a relationship between care provider volume and post-operative CC mortality [22]–[25], and are in contrast with other studies that failed to demonstrate such a relationship [26]–[28].

Few studies examined the relationship of care provider volume with re-intervention and re-admission after CC surgery, and evidence on this topic is mixed [25], [28], although these outcomes have been advocated as potentially useful targets for measurement of the quality of surgical care [17], [29]–[32]. In the present study, we did not observe any relationship of re-intervention and re-admission with surgical volumes.The re-admission rate in our study was 28%, which exceeds the range of rates from the literature (11–27%) [30]–[34]. However, this should be interpreted keeping in mind that our post-operative mortality is at the lower boundary of the mortality rate range reported in other studies (1–11%) [4]–[8], [13], [23], [25], [26], [28]. High rates of re-hospitalization may reflect a care policy favoring early detection and treatment of surgical complications with the aim of reducing the prevalence of post-operative deaths [35]. However, there is also evidence that unplanned 30-day re-admission may be associated with increased post-operative mortality [31].

Our results concerning the relationship between focused practice and outcomes indicate that GSUs with ≥5% CC over total surgery had significantly lower re-admission and re-intervention rates, but did not differ from GSUs with <5% CC on post-operative mortality rates. This suggests that studies on CC surgery outcomes should examine both the effects of volumes and focused practice, because these two variables have a different pattern of association with outcomes. Although evidence from the literature on the effect of focused practice is not available, a recent Spanish study carried out on patients undergoing emergency colorectal resection showed that being operated by a colorectal surgeon compared with a general surgeon was associated with a lower 30-day mortality, after adjusting for patients’ gender, age, ASA score and type of operation [36].

We found that urgent procedures were associated with a higher 30-day mortality. Specifically, urgent patients were about six times as likely to die within 30 days compared with elective patients. This finding is consistent with the literature, indicating that urgent procedures are strongly associated with adverse outcomes after colorectal resection [19], [37], [38], although these authors had a broader focus on colorectal surgery for any reason, and not only for CC. Furthermore, separate analyses carried out in urgent and elective patients revealed that the adjusted risk of post-operative mortality was increased in low-volume GSUs for elective CC surgery, but not for urgent surgery. We also found that more than 40% of the variability in 30-day mortality for elective surgery was accounted for by the GSU volume, whereas no significant variation was found among GSUs for urgent surgery. This is in contrast with the results of a recent study in Denmark, in which a significant variation in mortality between low- and high-volume hospitals was found for urgent (but not elective) surgery [19].

Our findings of better outcomes in high-volume settings bear directly to the question of whether GSU volume is a proxy of other variables such as availability of sophisticated clinical services (e.g., intensive care units (ICUs) and advanced diagnostic/interventional services) and high quality of nursing care. These variables have been proposed as explanatory variables of better outcomes, in particular mortality, in high-volume centers [17], [39]–[41].

The relationship between volumes and outcomes has substantial clinical and organizational implications. In fact, unlike the case of less frequent complex procedures in which the overall effect of higher GSU volumes makes centralization desirable, unintended negative consequences of centralizing colonic resection for cancer must be considered [15]. Referring a large number of cases to a limited number of centers might decrease accessibility for patients and their families [42], and threaten continuity of care after surgery.

However, because our results suggest a relationship between GSU volumes and outcomes in elective patients, we argue that centralization may facilitate the quality of surgery for these patients, including for screen-detected ones, to avoid exposure of apparently healthy people to unnecessary harmful treatments [43].

Our results should be interpreted keeping in mind some important limitations. First, administrative databases have a limited ability to capture illness severity. To minimize this bias, in the absence of information on cancer staging, we classified cancers as metastatic/non-metastatic. Moreover, we considered comorbidities in the index hospitalization and those of the two previous years, as suggested in Davoli *et al.* [44]. In this way associated relevant medical illnesses, that most likely affect outcomes, were taken into consideration. Second, the potential for inaccurate coding exists in administrative databases such as the hospital discharge records database. However, one study using administrative data in our region showed that hospital discharge records have good specificity, sensitivity and positive predictive value (84.8%, 99.0% and 90.6%), compared with cancer registries [45]. Moreover, the lack of ambiguity regarding diagnoses and procedures for CC, coupled with the fact that outcomes of interest are well defined and not particularly subject to misinterpretation, minimizes this potential bias. Third, we might have not captured the full spectrum of post-operative morbidity. However, many adverse events occurring after colonic resection are recognized during the index hospitalization. In this regard, we searched re-interventions related to index procedures using a specific subset of severe surgical complications. Moreover, we used the 30-day re-admission rate, that may be considered as a fairly good surrogate of surgical complications occurring after hospital discharge [31], [34]. Fourth, information on the individual surgeon volume is not available from administrative databases. Higher surgeon volumes were associated with better outcomes in several studies, including for instance Birkmeyer and Chang [46], [17]. Lastly, we could not examine the relationship of GSU volumes and other provider characteristics with other outcomes of CC surgery (e.g. radical nature of the resection, number of retrieved lymph nodes, local recurrence rate and disease free survival) which are decisive to monitor quality of care and focus improvement initiatives in CC surgery [18] because this information is not available in routine databases.

### Conclusions

The present study provided further evidence of the beneficial effect of GSU volume on mortality for elective CC surgery and of focused practice on re-intervention and re-admission. This indicates that clinicians, policy makers and hospital administrators should consider the opportunity to centralize CC surgery keeping in mind their pros and cons, and establish audit of current practice and outcomes to ensure that the benefits of high-volume and focused practice care can be translated into service organization.

## Supporting Information

Text S1 **ICD-9-CM diagnostic codes identifying surgical complications.**
(DOC)
