# Supplementary material for: Impact of Procedure Volumes and Focused Practice on Short-Term Outcomes of Elective and Urgent Colon Cancer Resection in Italy
Source: PLoS One. 2013 May 16;8(5):e64245. doi: 10.1371/journal.pone.0064245 (PMC3656123; doi:10.1371/journal.pone.0064245)
Supplement: Text S1 — ICD-9-CM diagnostic codes identifying surgical complications. (DOC) [file pone.0064245.s001.doc]

**TEXT S1.** ICD-9-CM diagnostic codes identifying surgical complications.

996–999 (Complications of surgical and medical care, not elsewhere classified), 536.4x (Gastrostomy complications), 537.4 (Fistula of the stomach or duodenum), 551.2x (Ventral hernia with gangrene), 552.2x (Ventral hernia with obstruction), 557.0 (Acute vascular insufficiency of intestine), 557.1 (Chronic vascular insufficiency of intestine), 557.9 (Unspecified vascular insufficiency of intestine), 560.xx (Intestinal obstruction without mention of hernia), 566 (Abscess of anal and rectal regions), 567.xx (Peritonitis and retroperitoneal infections), 568.0 (Peritoneal adhesions), 568.8x (Other specified disorders of peritoneum), 569.3 (Hemorrhage of rectum and anus), 569.6x (Colostomy and enterostomy complications), 569.81 (Fistula of intestine, excluding rectum and anus), 569.83 (Perforation of intestine), 569.85 (Angiodysplasia of intestine with hemorrhage), 569.86 (Dieulafoy lesion of intestine), 569.89 (Other [enteroptosis, granuloma of intestine, prolapse of intestine, pericolitis, perisigmoiditis, visceroptosis]), 570 (Acute and subacute necrosis of liver), 575.4 (Perforation of gallbladder), 575.5 (Fistula of gallbladder), 576.3 (Perforation of bile duct), 576.4 (Fistula of bile duct), 578.x (Gastrointestinal hemorrhage), 285.1 (Acute posthemorrhagic anemia).
